# Immuno and Affinity Cytochemical Analysis of Cell Wall Composition in the Moss *Physcomitrella patens*

**DOI:** 10.3389/fpls.2016.00248

**Published:** 2016-03-08

**Authors:** Elizabeth A. Berry, Mai L. Tran, Christos S. Dimos, Michael J. Budziszek, Tess R. Scavuzzo-Duggan, Alison W. Roberts

**Affiliations:** Department of Biological Sciences, University of Rhode IslandKingston, RI, USA

**Keywords:** affinity cytochemistry, bryophyte, cell wall, flow cytometry, immunofluorescence, *Physcomitrella patens*

## Abstract

In contrast to homeohydric vascular plants, mosses employ a poikilohydric strategy for surviving in the dry aerial environment. A detailed understanding of the structure, composition, and development of moss cell walls can contribute to our understanding of not only the evolution of overall cell wall complexity, but also the differences that have evolved in response to selection for different survival strategies. The model moss species *Physcomitrella patens* has a predominantly haploid lifecycle consisting of protonemal filaments that regenerate from protoplasts and enlarge by tip growth, and leafy gametophores composed of cells that enlarge by diffuse growth and differentiate into several different types. Advantages for genetic studies include methods for efficient targeted gene modification and extensive genomic resources. Immuno and affinity cytochemical labeling were used to examine the distribution of polysaccharides and proteins in regenerated protoplasts, protonemal filaments, rhizoids, and sectioned gametophores of *P. patens*. The cell wall composition of regenerated protoplasts was also characterized by flow cytometry. Crystalline cellulose was abundant in the cell walls of regenerating protoplasts and protonemal cells that developed on media of high osmolarity, whereas homogalactuonan was detected in the walls of protonemal cells that developed on low osmolarity media and not in regenerating protoplasts. Mannan was the major hemicellulose detected in all tissues tested. Arabinogalactan proteins were detected in different cell types by different probes, consistent with structural heterogneity. The results reveal developmental and cell type specific differences in cell wall composition and provide a basis for analyzing cell wall phenotypes in knockout mutants.

## Introduction

When compared to vascular plants, mosses employ a fundamentally different strategy for surviving in the dry aerial environment. Vascular plants are homeohydric with a cuticularized epidermis that resists water loss and vascular tissue to distribute water internally. In contrast, mosses are poikilohydric with aerial surfaces adapted to absorb water from their immediate surroundings and no true vascular tissue (Mishler and Oliver, 2009). In each case, cell walls serve as the boundary between the symplast and the external environment and confer special properties to different cell types. Thus, the cell walls of vascular plants and mosses have evolved in response to different selective pressures. A detailed understanding of the structure, composition and development of moss cell walls can contribute our understanding of not only the evolution of overall cell wall complexity, but also the differences that have evolved in response to selection for poikilohydric vs. homeohydric survival strategies (Roberts et al., 2012).

Establishment of *Physcomitrella patens* as a model moss species was fostered by its advantages for genetic studies, including methods for efficient targeted gene modification (Cove, 2005). Current investigations of gene function in *P. patens* are supported by genomic resources that include a sequenced genome (Rensing et al., 2008; Zimmer et al., 2013), full length cDNA clones (Nishiyama et al., 2003), and public microarray data (Cuming et al., 2007; Richardt et al., 2010; Hiss et al., 2014) for analysis of gene expression. *P. patens* genes that encode members of the glycosyl transferase families putatively responsible for biosynthesis of various cell wall polysaccharides have been identified by phylogenetic analysis (Roberts and Bushoven, 2007; Schuette et al., 2009; Yin et al., 2009, 2010; Harholt et al., 2012; Kulkarni et al., 2012; Hornblad et al., 2013; Jensen et al., 2014; McCarthy et al., 2014) and targeted gene modification approaches have the potential to reveal the functions of these proteins (Fu et al., 2007; Wise et al., 2011; Goss et al., 2012; Hornblad et al., 2013). Molecular probes provide one means to test for changes in the localization of specific cell wall structural motifs resulting from glycosyl transferase mutations.

Like all bryophytes, *P. patens* has a predominantly haploid lifecycle. The haploid phase consists of protonemal filaments that enlarge by tip growth (Menand et al., 2007) as well as leafy gametophores with several different cell types that enlarge by diffuse growth. Glycome profiling and carbohydrate linkage analysis revealed that *P. patens* cell walls contain many of the same components as *Arabidopsis* cell walls (Moller et al., 2007; Kulkarni et al., 2012) and some polymers, including arabinogalactan proteins (AGPs) (Fu et al., 2007), xyloglucan (Peña et al., 2008), and xylan (Kulkarni et al., 2012) have been analyzed structurally. A few focused studies have examined the distribution of specific polysaccharides, including xylan (Kulkarni et al., 2012), AGP (Lee et al., 2005a,b), callose (Schuette et al., 2009), mannan (Liepman et al., 2007; Lee et al., 2011), and cellulose (Goss et al., 2012). However, development related and cell type specific differences in cell wall composition have not been well characterized in *P. patens* or other mosses.

Here we report an analysis of *P. patens* gametophyte cell wall composition using monoclonal antibodies and carbohydrate binding modules (CBMs) in order to provide a basis for mutant analysis.

## Materials and Methods

### Probes

The probes used for labeling cell wall polysaccharides in *P. patens* were chosen based on an earlier Comprehensive Microarray Polymer Profiling (CoMPP) analysis (Moller et al., 2007) with some additions (**Table 1**). Antibodies included anti-homogalacturonan (HG) JIM5, JIM7, LM18, LM19, LM20 (Verhertbruggen et al., 2009), anti-1-4-β-D-galactan LM5 (Jones et al., 1997), anti-1-5-α-L-arabinan LM6 (Willats et al., 1998), anti-1-3-β-D-glucan BS400-4 (Meikle et al., 1991), anti-xylan LM10 (McCartney et al., 2005), anti-xyloglucan LM15 (Marcus et al., 2008), anti-mannan BS400-4 (Pettolino et al., 2001), and anti-AGP LM2 (Smallwood et al., 1996) and JIM13 (Knox et al., 1991). CBMs used for labeling included CBM3a and CBM28 (Blake et al., 2006). Anti-extensin probes were not tested based on lack of cross-reactivity shown by CoMPP (Moller et al., 2007). Antibodies designated JIM and LM, along with CBM3A, were obtained from Plant Probes (Leeds, UK) and antibodies designated BS were obtained from Australian Biosupplies (Bundoora, VIC, Australia). CBM28 was a gift of Paul Knox (University of Leeds). Other antibodies used included Alexafluor 488-conjugated anti-mouse and anti-rat (Life Technologies, Grand Island, NY, USA) and mouse anti-His (Sigma–Aldrich, St. Louis, MO, USA).

**Table 1:** Summary of antibody and CBM labeling of *Physcomitrella patens* tissues.

Probe	Epitope	CoMPP (Moller et al., 2007)	Protoplast	Protonema	Rhizoid	Gametophore
**Glucans**						
CBM3a	Cellulose, crystalline	YES	YES	YES	YES	YES
CBM28	Cellulose, non-crystalline	NT	YES	ND	ND	ND
BS 400-2	Callose	YES	YES	YES	YES	YES
**Hemicellulose**						
LM10	Xylan, low-substituted	YES	ND	ND	ND	YES
LM15	Xyloglucan, non-fucosylated	YES	YES	YES	ND	ND
BS 400-4	Mannan and glucomannan	YES	YES	YES	YES	YES
**Pectin**						
JIM5	Homogalacturonan, +/− methyl-esterified	YES	NT	NT	NT	YES
LM18	Homogalacturonan, +/− methyl-esterified	NT	ND	NT	NT	YES
LM19	Homogalacturonan, unesterified	NT	ND	YES	ND	YES
JIM7	Homogalacturonan, methyl-esterified only	YES	NT	NT	NT	YES
LM20	Homogalacturonan, methyl-esterified only	NT	ND	NT	ND	ND
LM5	1,4-β-D-galactan	YES	YES	NT	ND	YES
LM6	1,5-α-L-arabinan	YES	YES	YES	YES	YES
**Glycoproteins**						
LM2	Arabinogalactan protein	YES	YES	YES	YES	YES
JIM13	Arabinogalactan protein	YES	YES	NT	ND	YES

*ND, none detected; NT, not tested.*

### *Physcomitrella patens* Culture

Protoplasts were prepared from *P. patens* Gransden (Rensing et al., 2008) as described previously (Roberts et al., 2011) and suspended in liquid protoplast regeneration medium (PRML) at a density of 50,000 cells mL^–1^. Plates containing solid protoplast regeneration medium (PRMB) overlain with cellophane were inoculated with 1 mL of protoplast suspension and incubated for 24 h at 25^o^C with constant illumination at 50–80 μmol m^–2^. For regeneration of protonemal filaments, protoplasts were isolated as described above, plated at a rate of 15,000 cells plate^–1^, and incubated as above for 48 h. Cellophane disks with developing filaments were then transferred to basal medium supplemented with ammonium tartrate (BCDAT) and incubated as above for 48 h. Some cultures were then fixed immediately and others were transferred back to PRMB medium and incubated as above for 48 h before fixation (Roberts et al., 2011). Gametophores were harvested from tissue clumps cultured on BCDAT medium without cellophane for 3–4 weeks. Rhizoid development was stimulated by culturing tissue clumps on BCD medium supplemented with 1 μM naphthalene acetic acid (Sakakibara et al., 2003).

### Specimen Preparation

Protoplasts cultured for 24 h were collected by washing the plates with 3 mL of de-ionized water. After determining cell number using a hemocytometer, the protoplasts were collected by centrifugation in a clinical centrifuge at speed 4, with no braking, for 3 min. The resulting pellet was resuspended in 1 mL of fixative (7% w/v formaldehyde, 50 mM PIPES, pH 6.8, 2.5 mM magnesium sulfate, 5 mM EGTA) for either 20 min at room temperature or overnight at 4°C. Fixed protoplasts were subjected to three washes that included centrifugation for 3 min at speed 4 with a clinical centrifuge and resuspension in 3 mL of phosphate-buffered saline (PBS). 100,000 protoplasts were aliquoted into 1.5 mL tubes, resuspended in 200 μL blocking solution (5% w/v non-fat dry milk in PBS) for 20 min, and collected by centrifugation at 1000 x *g* for 5 min. Protoplasts were then labeled with either (1) LM or JIM antibodies diluted 1:5 in blocking solution for 1.5 h followed by labeling with anti-rat IgG AlexaFluor488 (Life Technologies) diluted 1:50 in blocking solution for 1 h, or (2) labeled with BS400-2 or BS400-4 diluted 1:200 in blocking solution for 1.5 h followed by labeling with anti-mouse IgG AlexaFluor488 (Life Technologies) diluted 1:50 in blocking solution for 1 h, or (3) labeled with CBM3a or CBM28 (5 μg ml^–1^) in blocking solution for 1 h followed by labeling with mouse anti-polyhistidine (Sigma–Aldrich) diluted 1:100 in blocking solution for 1.5 h and anti-mouse IgG AlexaFluor488 secondary antibody diluted 1:50 in blocking solution for 1 h. After each labeling step, protoplasts were subjected to three washes that included centrifugation at 1000 x *g* for 5 min and resuspension in 500 μL of PBS. 10 μL of suspension was mounted on a glass slide with Prolong Gold anti-fade mounting reagent (Life Technologies) for imaging and the rest was analyzed by flow cytometry.

Protonemal filaments were collected from Petri plates, fixed, mounted on slides, and labeled with antibodies or CBMs as described previously (Roberts et al., 2011). Rhizoids were collected from 14-day-old cultures and fixed and labeled as described for protoplasts.

Whole gametophores were collected with forceps and immersed in fixative (25 μM Na phosphate, pH 7.1, 1.6% w/v formaldehyde, 0.2% w/v glutaraldehyde). Following fixation for 1 h at 21^o^C or overnight at 4^o^C, gametophores were dehydrated through an ethanol series and infiltrated with LR White resin (Kulkarni et al., 2012). Sections (1–2 μm) were cut with glass knives, mounted on slides, and labeled with antibodies as described previously (Kulkarni et al., 2012). For CBM labeling, the blocking step was followed by incubation for 2 h at RT in CBM (5 μg ml^–1^ in blocking solution), washing (Kulkarni et al., 2012), and incubation with anti-polyhistidine (Sigma, 1:100 in blocking solution) in place of primary antibody.

Specimens were examined using a BHS microscope with blue filter cube (Olympus, Center Valley, PA, USA) and images were captured using either an RT Slider camera (Spot Imaging, Sterling Heights, MI, USA) in monochrome mode or a DFC310FX color camera (Leica Microsystems Inc., Buffalo Grove, IL, USA). For all labeling experiments, controls were prepared without primary antibody or without CBM. Controls were photographed with the same microscope and manual camera settings to distinguish autofluorescence and non-specific secondary antibody binding from specific labeling.

### Flow Cytometry

Labeled protoplasts were analyzed using a BD Influx flow cytometer with 100 μm flow tip, FACS sheath fluid, and FACS Software V1.0 (BD Bioscience, San Jose, CA, USA). Flow rates were set to approximately 200 cells per second. Voltages were set to gate negative control (protoplasts with no primary antibody staining) and protoplasts with the highest AlexFluor488 fluorescence (stained with CBM3a) within the 530/40 plotting range. Approximately 30,000 events were collected per sample. Population 1 was selected based on forward scattering (FSC) and side scattering (SSC) for round protoplasts (Harkins and Galbraith, 1984). Population 1 was examined for chlorophyll autofluorescence with a 692/40 filter, which revealed two populations of protoplasts with high and low intensities for chlorophyll autofluorescence. Population 2 with high chlorophyll autofluorescence and population 3 with low chlorophyll autofluorescence were gated separately and measured for fluorescent intensity of AlexaFluor488 with a 530/40 filter. All experiments were repeated in duplicate with three pooled biological replicates. *T*-test was used to test for statistical significance between high and low chlorophyll mean fluorescence intensity for each probe and mean fluorescence intensity between positive and negative staining.

## Results

### Cell Wall Composition of Regenerating Protoplasts

**FIGURE 1:**
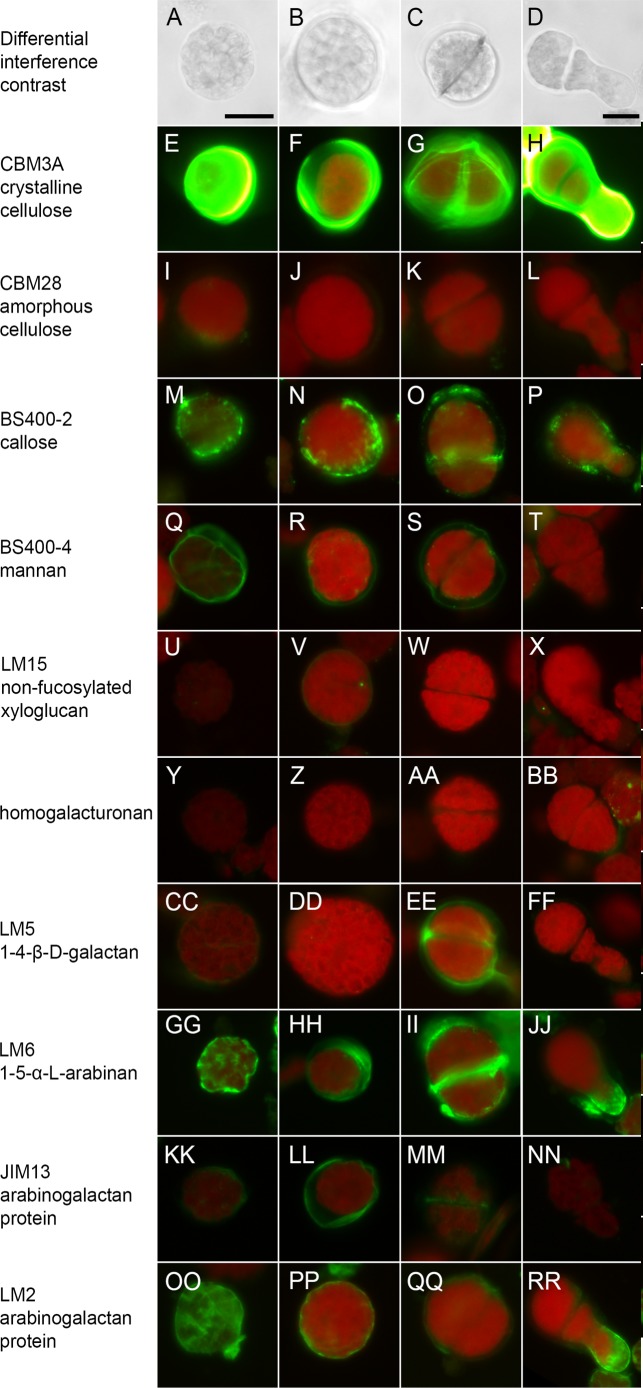
**Protoplasts of ***Physcomitrella patens*** cultured for 24 h on protoplast regeneration medium and labeled with carbohydrate binding modules (CBMs) or antibodies**. Differential interference contrast images show four stages of regeneration including **(A)** thin-walled stage, **(B)** thick-walled stage, **(C)** divided, and **(D)** filament extension and head columns of images of cells at the same stage and labeled with different probes. Protoplasts were labeled with **(E–H)** CBM3a for crystalline cellulose, **(I–L)** CBM28 for non-crystalline cellulose, **(M–P)** anti-callose (BS-400-2), **(Q–T)** anti-mannan (BS-400-4), **(U–X)** anti-xyloglucan (LM15), **(Y–BB)** anti-HG (LM19 in **Y-AA**, LM20 in **BB**), **(CC–FF)** anti-1,4-β-D-galactan (LM5), **(GG–JJ)** anti-1,5-α-L-arabinan (LM6), **(KK–NN)** anti-arabinogalactan protein (JIM13), and **(OO–RR)** anti-arabinogalactan protein (LM2) Bars = 20 μm.

**FIGURE 2:**
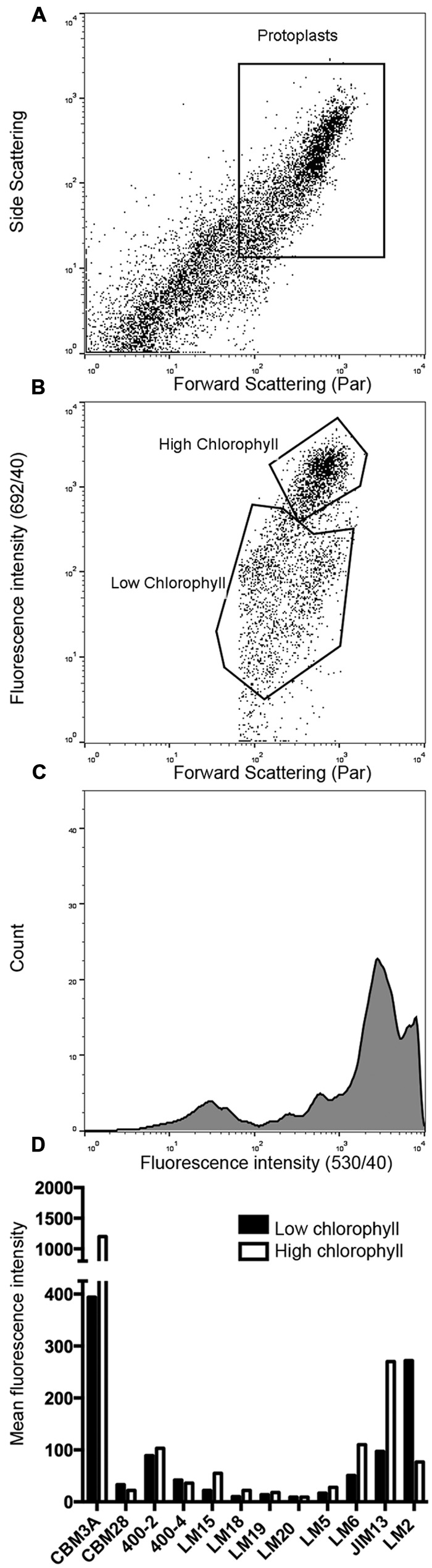
**Flow cytometry analysis of ***P. patens*** protoplasts regenerated for 24 h and labeled with CBM or antibodies. (A)** Scatter plot of protoplasts labeled with CBM3a and gated for roundness based on forward scatter (FSC) and side scatter (SSC). **(B)** Round protoplasts (population 1 above) gated for high and low chlorophyll autofluorescence. **(C)** Histogram of fluorescence intensities of population 1 showing peaks for subpopulations with high and low chlorophyll autofluorescence. **(D)** Mean fluorescence intensities (530/40) of regenerated protoplasts gated for roundness based on FSC and SSC and then for low vs. high chlorophyll autofluorescence. Differences in fluorescence intensity between high and low chlorophyll populations were significant (*p* = < 0.003) for all probes except LM18, LM19, and LM20, which were not significantly greater than the negative control.

To identify the polysaccharide components that are deposited in the course of cell wall regeneration, protoplasts cultured for 24 h in PRML were labeled with antibodies or CBMs for fluorescence microscopy and fluorescence quantification by flow cytometry. Four different stages of protoplast regeneration were observed by fluorescence microscopy in the fixed and processed suspensions. Most cells were spherical and had either thin cell walls and weak chlorophyll autofluorescence (**Figure 1A**) or thick cell walls and strong chlorophyll autofluorescence (**Figure 1B**). A small proportion (<1%) of cells had divided (**Figure 1C**) or divided and begun to regenerate a protonemal filament (**Figure 1D**). In flow cytometry analysis (**Figure 2**), populations of protoplasts were selected for roundness based on FSC and SSC and cellular debris, which has very low FSC and SSC, was omitted from the analysis (**Figure 2A**). As expected, negative controls had no or very low fluorescence with mean fluorescent intensities of less than 20 and served as a baseline for the measurement of fluorescent intensity. Round protoplasts had a wide range of chlorophyll autofluorescence (692/40) and could be divided into populations with high and low autofluorescence on the scatter plot of FSC vs. 692/40 (**Figures 2B,C**). While developing a cell wall permeabilization method for immunofluorescent labeling of tubulin in cultured cells (Uhnak and Roberts, 1995), we noted an inverse relationship between chlorophyll autofluorescence and antibody penetration, leading us to conclude that the cell wall can block chlorophyll extraction during processing. The difference in chlorophyll autofluorescence between thin-walled and thick-walled cells observed by fluorescence microscopy in regenerating *P. patens* protoplasts was consistent with this interpretation. Thus, we gated populations with high and low chlorophyll fluorescence as a means of comparing labeling (530/40) of cells in the early and later stages of cell wall regeneration.

Based on strong labeling with CBM3a, the cell walls of protoplasts at all stages of regeneration contain a high proportion of crystalline cellulose (**Figures 1E–H**). In contrast, a probe for non-crystalline cellulose (CBM28) showed little or no labeling (**Figures 1I–L**), indicating that the cellulose in regenerated cell walls is primarily in the crystalline form. These results were corroborated by flow cytometry, which showed strong fluorescence for CBM3a with significantly more labeling in the high chlorophyll autofluorescence population and weak labeling with CBM28 (**Figure 2D**). Anti-callose (BS 400-2) labeled all regenerated cell walls in a punctate pattern (**Figures 1M–P**) and also localized to the cell plate in dividing cells (**Figure 1O**). This is consistent with moderate levels of anti-callose labeling detected by flow cytometry with slightly higher labeling in high chlorophyll autofluorescence populations (**Figure 2D**).

Of the hemicellulose probes tested, only anti-mannan (BS 400-4) showed detectable labeling of thin cell walls and it also weakly labeled thicker cell walls (**Figures 1Q–T**). Weak labeling was observed with anti-xyloglucan (LM15) only in undivided cells with thicker cell walls (**Figures 1U–X**). Similarly, flow cytometry detected low levels of anti-mannan labeling with higher levels in low chlorophyll autofluorescence populations and weak anti-xyloglucan labeling with higher levels in the high chlorophyll autofluorescence population (**Figure 2D**). Anti-xylan (LM10) did not label regenerating protoplasts in preliminary experiments and was not further tested.

No labeling with anti-HG (LM18, LM19, and LM20) was detected in the protoplasts with fluorescence microscopy (**Figures 1Y–BB**) or flow cytometry (**Figure 2D**). However, labeling with probes for the pectin epitopes 1,4-β-D-galactan (LM5, **Figures 1CC–FF**) and 1,5-α-L-arabinan (LM6, **Figures 1GG–JJ**) was detected by fluorescence microscopy. Labeling of 1,5-α-L-arabinan and 1,4-β-D-galactan was also detected by flow cytometry with higher levels in the high chlorophyll autofluorescence population (**Figure 2D**). This is consistent with labeling of only a very small number of thick-walled cells (not shown) and dividing cells (**Figure 1EE**) with anti-1,4-β-D-galactan as detected by fluorescence microscopy.

The antibodies LM2 and JIM13 showed the strongest labeling of any probe except CBM3A (**Figures 1K–RR** and **2D**), indicating that the cell walls of regenerating protoplasts are rich in AGP. We also noted differences in the labeling patterns of JIM13 (**Figures 1KK–NN**) and LM2 (**Figures 1OO–RR**), which recognize different AGP epitopes. Whereas JIM13 labeling predominated in the high chlorophyll population LM2 labeling was greater in the low chlorophyll population (**Figure 2D**). Like LM6 (**Figure 1JJ**), LM2 strongly labeled the tips of emerging protonemal filaments (**Figure 1RR**).

### Cell Wall Composition of Protonemal Filaments

**FIGURE 3:**
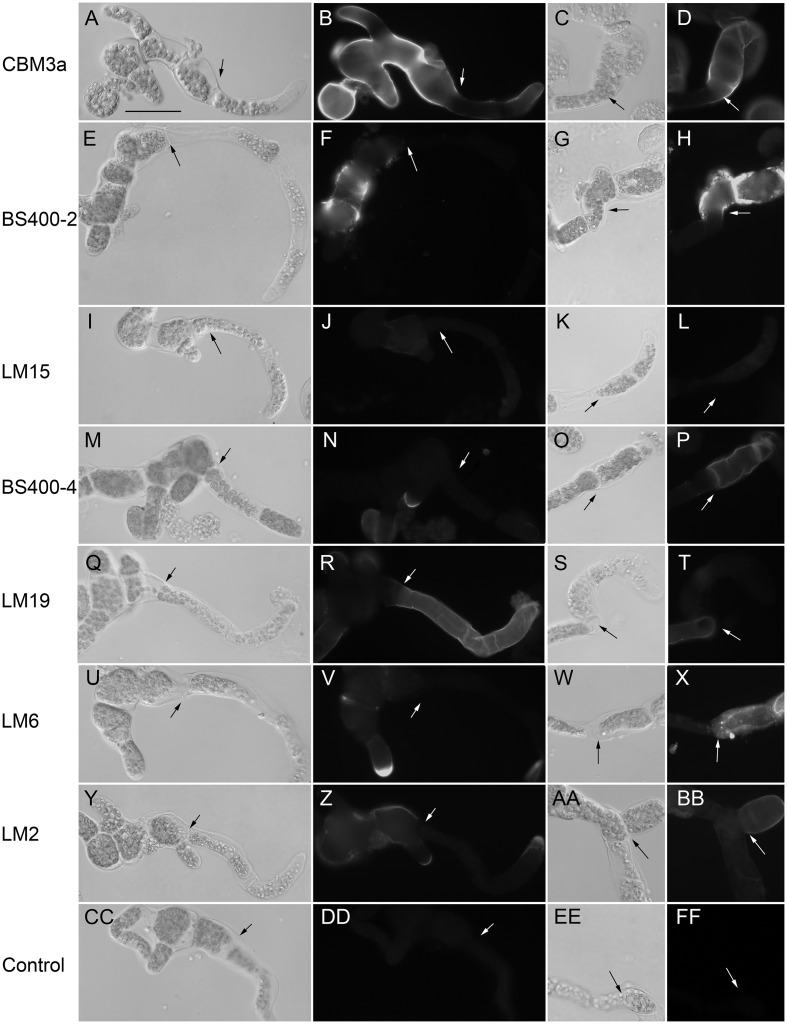
***Physcomitrella patens* protonemal filaments regenerated from protoplasts and labeled with antibodies or CBMs**. Protoplasts were fixed after regeneration on medium containing mannitol for 2 days and growth on mannitol-free medium for 2 days (columns 1 and 2) or after the same treatment followed by two additional days growth on medium containing mannitol (columns 3 and 4). Colonies were imaged with DIC optics (columns 1 and 3) or fluorescence optics (columns 2 and 4) after labeling with **(A–D)** CBM3a for crystalline cellulose, **(E–H)** anti-callose (BS-400-2), **(I–L)** anti-xyloglucan (LM15), **(M–P)** anti-mannan (BS-400-4), **(Q–T)** anti-HG (LM19), **(U–X)** anti-1,5-α-L-arabinan (LM6), **(Y–BB)** anti-arabinogalactan protein (LM2) or no primary antibody **(CC–FF)**. Arrows indicate the transition between cells that developed from protoplasts on mannitol-containing medium (left) and cells that developed on mannitol-free medium (right) in column 1 and 2 and between cells that developed on mannitol-free medium (left) and after transfer to mannitol-containing medium (right) in columns 3 and 4. Bar = 50 μm.

After culturing for 48 h on PRMB, which contains 6% w/v mannitol as an osmoticum, regenerated protoplasts consisting of 2–3 cells are routinely transferred to standard culture medium (BCDAT) for further development (Cove et al., 2009). Initial examination of 4-d-old colonies derived from protoplasts regenerated for 48 h on PRMB then cultured for 48 h on BCDAT (**Figures 3A,B**) revealed strong CBM3a labeling of the original protoplast and adjacent cells that had deposited cell walls in the presence of mannitol. In contrast, the cells near the tips of the filaments that had deposited cell walls in the absence of mannitol were weakly labeled except for the very tips of the apical cells. In addition to strong cell wall labeling with CBM3a, the cells that developed in the presence of mannitol were shorter and thicker (**Figures 3A,B**). To test whether these morphological and cell wall compositional differences were due to regeneration from protoplasts or growth on mannitol, the 4-d-old colonies were transferred back to PRMB for 48 h prior to labeling with CBM3a. The resulting 6-d-old colonies consisted of 6 or more cells and had begun to branch (**Figures 3C,D**). The cells closest to the tips were short, thick and labeled strongly with CBM3a, similar to the original protoplasts and adjacent cells. In contrast, the intervening cells were longer, thinner and stained weakly with CBM3a. Given that subapical cells do not enlarge after they are cut off from the apical cell by cytokinesis (Menand et al., 2007), this indicates that high osmolarity stimulates cellulose deposition in enlarging protonemal cells of *P. patens*.

To examine the effect of high osmolarity on the deposition of other cell wall polysaccharides, 4-d-old colonies that had been cultured from protoplasts for 2 days on PRMB and 2 days on BCDAT, and 6-d-old colonies that had been cultured from protoplasts for 2 day on PRMB, 2 days on BCDAT, and 2 days on PRMB were labeled with 10 additional probes. LM1, LM8, LM10, and CBM28 showed no fluorescence above negative control levels in which the primary antibody was omitted (**Figures 3CC–FF**), whereas 400-2, LM15, 400-4, LM19, LM6, and LM2 labeled the protonemal cell walls. Similar to CBM3a labeling, anti-1,3-β-glucan (BS400-2) strongly labeled the cell walls deposited in the presence of mannitol with little or no labeling of cell walls deposited in the absence of mannitol (**Figures 3E–H**). The pattern of anti-xyloglucan (LM15) labeling was similar, but overall labeling was much weaker compared to CBM3a and BS400-2 (**Figures 3I–L**). Anti-1,4-β-mannan (BS400-4) did not label the initial protoplast (**Figures 3M,N**), but did label the tips of filaments grown in the absence of mannitol and the cell walls deposited after transfer from mannitol-free to mannitol-containing medium (**Figures 3O,P**), with little labeling of the walls deposited in the absence of mannitol. In contrast to all other probes tested, anti-HG (LM19) labeled only the cell walls deposited in the absence of mannitol (**Figures 3Q–T**). The anti-1,5-α-L-arabinan (LM6) labeling pattern was similar to that of BS400-4 with the strongest fluorescence in the tips of filaments grown in the absence of mannitol and cell walls deposited after transfer from mannitol-free to mannitol-containing medium (**Figures 3U–X**). Anti-AGP (LM2) labeled cell walls deposited during protoplast regeneration and after transfer from mannitol-free to mannitol containing medium (**Figures 3Y–BB**), similar to CBM3a, BS400-2, and LM15.

### Cell Wall Composition of Rhizoids

**FIGURE 4:**
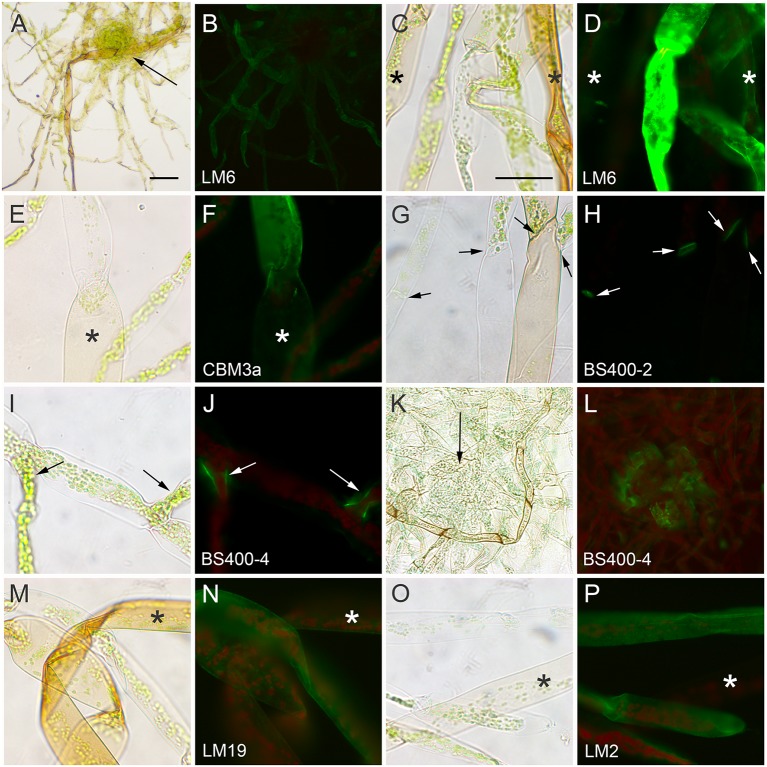
*****Physcomitrella patens*** rhizoids cultured on medium containing auxin (1 μM naphthalene acetic acid) for 2 weeks, fixed and labeled with antibodies or CBMs**. Rhizoids were imaged with bright field **(A,C,E,G,I,K,M,O)** and fluorescence optics **(B,D,F,H,J,L,N,P)** after labeling with **(A–D)** anti-1,5-α-L-arabinan (LM6), **(E,F)** CBM3a for crystalline cellulose, **(G,H)** anti-callose (BS-400-2), which labels cross walls indicated by arrows, **(I–L)** anti-mannan (BS-400-4), which labels cell junctions indicated by arrows, **(M,N)** anti-HG (LM19), and **(O,P)** anti-arabinogalactan protein (LM2). Mature pigmented rhizoids are indicated with asterisk. Bar = 200 μm in **(A)** for **(A,B)**. Bar = 100 μm in **(C)** for **(C–P)**.

Rhizoids typically develop from the gametophore axis and are initially obscured by leaves. Supplementing the culture medium with auxin enhances rhizoid initiation and inhibits leaf initiation (Sakakibara et al., 2003) allowing examination of rhizoids emerging from a leafless axis (**Figure 4A**). Young rhizoids are colorless, but accumulate brown pigments later in development (**Figure 4**). The radial walls of colorless rhizoids labeled strongly with anti-1,5-α-L-arabinan (LM6, **Figures 4C,D**), and weakly with CBM3a (crystalline cellulose, **Figures 4E,F**), anti-HG (LM19, **Figures 4M,N**), and anti-arabinogalactan protein (LM2, **Figures 4O,P**). The radial walls of pigmented rhizoid did not label with any of the probes tested (**Figure 4**). Anti-callose (BS-400-2) labeled only cross walls in pigmented and non-pigmented rhizoids (**Figures 4G,H**, arrows). Anti-mannan (BS-400-4) labeled radial walls, but only adjacent to branch points (**Figures 4I,J**, arrows) or where rhizoids emerged from the axis (**Figures 4K,L**, arrows). No labeling of rhizoids above negative control levels was detected with LM5, LM10, LM15, LM20, JIM13, and CBM28.

### Cell Wall Composition of Gametophores

**FIGURE 5:**
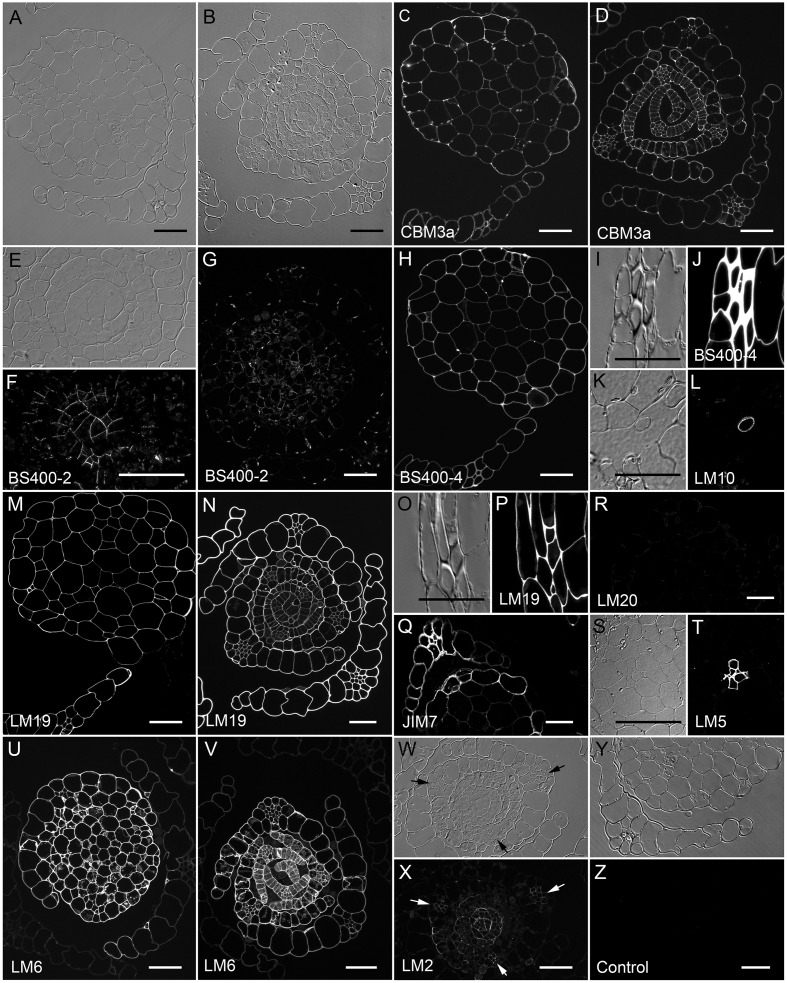
*****Physcomitrella patens*** gametophores labeled with antibodies or CBMs**. Transverse sections through mature stems and leaves **(A,C,G,H,M,Q,R,Y,Z)** and meristems and young leaves **(B,D,E,F,N,V,W,X)** imaged with DIC optics **(A,B,E,I,K,O,S,W,Y)** and with fluorescence optics after labeling for **(C,D)** crystalline cellulose with CBM3a, **(F,G)** callose with BS400-2, **(H,J)** mannan with BS400-4, **(L)** xylan with LM10, homogalacturonon with **(M,N,P)** LM19, **(Q)** JIM7 or **(R)** LM20, **(T)** 1,4-β-galactan with LM5, **(U,V)** 1,5-α-L-arabinan with LM6, **(X)** arabinogalactan proteins with LM2, or **(Z)** no primary antibody control. Higher magnification views show labeling of **(E,F)** cell plates with anti-callose, **(I,J)** the thick walls of stereid cells with anti-HG, **(K,L)** axillary hairs with anti-xylan, **(O,P)** the middle lamella of stereid cells with anti-homogalaturonon and **(S,T)** hydroids. All fluorescence images were captured using identical exposure conditions with the exceptions of **(L,Q,R)**, which were captured using a different microscope. Bars = 50 μm.

Sections of mature stems (**Figure 5A**) and apical regions (**Figure 5B**) of leafy gametophores were labeled to detect cell type and developmental difference in cell wall composition. Based on labeling with CBM3A, crystalline cellulose is present in the walls of all gametophore cells (**Figure 5C**) and more abundant in unexpanded cells of immature leaves (**Figure 5D**). A probe for callose (BS 400-2) labeled cell plates and the most recently deposited walls of meristem cells (**Figures 5E,F**). Punctate staining with BS 400-2 was noted in the cell walls of mature tissues (**Figure 5G**).

Of the hemicellulose probes tested, only anti-mannan showed strong labeling of all gametophore cell walls (**Figure 5H**), including the thick walls of the stereids that form the leaf midribs (**Figures 5I,J**). Anti-xylan (LM10) labeled only the walls of axillary hair cells (**Figures 5K,L**). A probe for non-fucosylated xyloglucan (LM15) did not label the walls of any gametophore leaf or stem cells (not shown).

The HG probe LM19 labeled the walls of all gametophore cells with relatively strong labeling of maturing leaves (**Figures 5M,N**). In contrast to anti-mannan labeling, LM19 labeling was restricted to middle lamella in the stereids (**Figures 5O,P**). Two probes for methyl-esterified HG gave different results; whereas JIM7 labeled all gametophore cell walls (**Figure 5Q**), no labeling was detected with LM20 (**Figure 5R**). Although labeling of tobacco sections with LM20 was enhanced by pre-treating with *N*-cyclohexyl-3-aminopropanesulfonic acid (CAPS) buffer at pH 9.5 (Verhertbruggen et al., 2009), this was not the case for *P. patens* (not shown). A probe for 1,4-β-galactan (LM5) specifically labeled the cell walls of hydroids with no labeling detected in the stereids of the leaf midribs or any other cell type (**Figures 5S,T**). The LM6 probe for 1,5-α-L-arabinan showed the strongest labeling of cell walls in mature stems (**Figure 5U**) and meristems (**Figure 5V**) and weak labeling of the mature leaves (**Figures 5U,V**).

One probe for AGP (LM2) labeled the walls of cells within the meristem (**Figures 5W,X**). As shown in cross sections through the apical region (**Figures 5W,X**), staining with LM2 diminished early in leaf development, except for the cells of the midribs, where it persisted until the leaves began to mature. No consistent cell wall labeling of gametophore sections was detected with AGP probe LM13 (not shown). With the exposure conditions used, no fluorescence was detected in sections that were labeled only with secondary antibody (**Figures 5Y,Z**). The results indicate that cell wall composition varies among the different cell types of the *P. patens* gametophore.

## Discussion

Chemical analyses have shown that *P. patens* cell walls contain structurally distinct forms of the same polysaccharides that are found in seed plant cell walls (Lee et al., 2005b; Fu et al., 2007; Moller et al., 2007; Peña et al., 2008; Kulkarni et al., 2012). Although originally designed to detect and localize cell wall polysaccharides in angiosperms, antibody and CBM probes have been used effectively to reveal structural heterogeneity in the cell walls of seedless vascular plants (Knox, 2008a; Harholt et al., 2012; Leroux et al., 2015), algae (Carafa et al., 2005; Domozych et al., 2007; Popper, 2008; Sørensen et al., 2011) and mosses, including *P. patens* (Ligrone et al., 2002; Kremer et al., 2004; Carafa et al., 2005; Lee et al., 2005a,b, 2011; Liepman et al., 2007; Schuette et al., 2009; Goss et al., 2012; Kulkarni et al., 2012). However, interpretation of labeling patterns and the presence or absence of polysaccharide classes must take into account potentially confounding factors including variation in the glycan epitopes associated with recognized classes of polysaccharides (Knox, 2008b), masking of scarcer epitopes by abundant polysaccharides (Marcus et al., 2008; Hervé et al., 2011; Xue et al., 2013), and the complex and, in some cases, incompletely characterized binding properties of the available probes (Willats and Knox, 2003). Despite the limitations, labeling with these probes has revealed spatial and developmental regulation of cell wall composition at the tissue, cell, and cell wall microdomain level in *P. patens*.

### Glucans

Based on labeling with CBM3a, crystalline cellulose is a component of all *P. patens* cell walls and is especially abundant in regenerating protoplasts and protonemal cell walls deposited after transfer of colonies to protoplast regeneration medium containing 6% w/v mannitol. This indicates that *P. patens* may up-regulate cellulose deposition in response to osmotic stress. In contrast, vascular plants down-regulate cellulose deposition when exposed to osmotic stress (Iraki et al., 1989a; Skirycz et al., 2010; Wang et al., 2016). These divergent responses may have evolved as a result of different selective pressures related to poikilohydric vs. homeohydric strategies. Non-crystalline cellulose was detected with CBM28 only in regenerating protoplasts and may have been deposited during the initial stages of cell wall regeneration.

Callose was the second most abundant polysaccharide in regenerating protoplasts and was also abundant in protonemal cell walls deposited in the presence of mannitol. Callose has been observed in the regenerated cell walls of protoplasts isolated from diverse plants (Shea et al., 1989). Although wound callose may be deposited in response to enzymatic digestion of the cell wall or protoplast isolation (Shea et al., 1989), deposits distinct from wound callose have also been observed in regenerated cell walls (van Amstel and Kengen, 1996). Callose labeling was also detected in cell plates in dividing protoplasts, within the gametophore meristem, and in rhizoid cross walls, consistent with previous observations in vascular plants (Gunning, 1982; Samuels et al., 1995). In another bryophyte, the liverwort *Marchantia polymorpha*, callose was observed in both regenerated walls and cell plates of protoplasts (Shibaya and Sugawara, 2009). In *P. patens*, *c*allose has been localized to the aperture exine in developing spores and parallels were drawn with sporogenesis in seed plants. The same study (Schuette et al., 2009) identified 12 *P. patens Callose Synthase* genes, consistent with multiple roles for callose in development and stress response. Overall, the developmental and stress-related roles of callose (Chen and Kim, 2009) appear to be conserved in mosses and seed plants.

### Hemicellulose

Among hemicelluloses, mannan appeared to be the most abundant in all tissues tested. This is consistent with relatively high levels of 4-linked mannan detected by chemical analysis (Moller et al., 2007). In protonemal filaments and rhizoids, mannan was localized at cell junctions consistent with previous observations for protonema (Liepman et al., 2007; Lee et al., 2011). Low levels of non-fucosylated xyloglucan were detected in protoplasts with LM15. Weak labeling with LM15 was also noted in protonemal filaments, consistent with LM15 results from CoMPP (Moller et al., 2007). No labeling was detected in gametophore cross sections or rhizoids This is in contrast to strong labeling observed with the CCRC-M88 xyloglucan probe (Kulkarni et al., 2012). Whereas LM 15 recognizes xyloglucan with the XXXG motif (Marcus et al., 2008), CCRC-M88 recognizes tomato xyloglucan, which is rich in the XXGG motif (Pattathil et al., 2010). The xyloglucan extracted from gametophores of *P. patens* is based on an XXGGG motif (Peña et al., 2008). Thus, labeling of *P. patens* cell walls with CCRC-M88, but not LM15, is not unexpected. Both branched and unbranched xylan was detected at low levels by CoMPP (Moller et al., 2007). Consistent with previous reports using LM11 (Kulkarni et al., 2012), we observed xylan labeling with LM10 mainly in axillary hairs.

### Pectin

Low levels of HG have been detected in *P. patens* protonemal tissue by CoMPP using antibodies JIM5, JIM7, and mAb2F4 (Moller et al., 2007). It has also been observed that JIM5 labels protonemal cell walls (Lee et al., 2005b). Here JIM5 and JIM7, as well as with LM18, LM19, and LM20 (Verhertbruggen et al., 2009), were used in an effort to distinguish methyl-esterified and unesterified HG. Probes that bind preferentially to de-esterified HG (LM18, LM19, and JIM5) (Verhertbruggen et al., 2009) labeled all cell types in gametophore sections. Using LM19, HG was detected in protonemal cell wall deposited in the absence of osmotic stress, but not in protoplasts or rhizoids. JIM7, which recognizes only methyl-esterified HG (Verhertbruggen et al., 2009), also labeled all cell types in gametophores. However, LM20, which also recognizes methyl-esterified HG, did not label gametophores, protoplasts, or rhizoids. Labeling with LM20 was also not enhanced by pre-treating section with CAPS buffer at pH 9.5 in contrast to previous reports for tobacco stem pith sections (Verhertbruggen et al., 2009). LM20 also failed to label *P. patens* gametophore cell walls as detected by TEM (Mansouri, 2012). Although the binding properties of JIM7 and LM20 show many similarities, differences in labeling patterns of tobacco stem pith cell walls indicate that they recognize different epitopes (Verhertbruggen et al., 2009). Because these epitopes have not been defined, we are unable to draw conclusions about the HG structure of *P. patens* cell walls based on differential labeling with JIM7 and LM20. However, labeling with LM18, LM19, and JIM7 indicate that HG is a component of *P. patens* protonemal and gametophore cell walls.

The rhamnogalacturonan (RGI) epitopes 1,5-α-L-arabinan (LM6) and 1,4-β-D-galactan (LM5) were detected in protoplasts and LM6 also labeled protonemal cell wall deposited in the presence of mannitol. In contrast to the response of cellulose deposition noted above, *P. patens* responds similarly to flowering plants in the effect of osmotic stress on pectin deposition. In both flowering plants (Iraki et al., 1989b) and *P. patens*, exposure to osmotic stress results in reduced HG deposition and enhanced RGI deposition. Besides protoplasts, LM5 labeled only hydroids in gametophore cross-sections. This is consistent with the results of a TEM study of *P. patens* meristems that showed specific LM5 labeling of the central strand cells, which are the precursors of hydroids (Mansouri, 2012) and indicates that hydroid cell walls have a distinct composition. In contrast, LM6 labeled protonema, rhizoid and gametophore cell walls, in addition to regenerating protoplasts. The presence of RGI in gametophore cell walls is also supported by labeling with CCRC-M35, a probe for the RGI backbone (Kulkarni et al., 2012).

### Arabinogalactan Proteins

Based on previous CoMPP analysis (Moller et al., 2007), *P. patens* cell walls do not contain epitopes recognized by the MAC207 and JIM8 probes for type 1 AGP (Knox et al., 1991). The charophycean green alga *Micrasterias denticulata* has also been reported to lack type I AGP (Eder et al., 2008). The LM2 probe for type II AGP labeled cell walls of developing protoplasts, growing protonemal tips, developing rhizoids, and the meristematic regions and developing midribs of gametophores in *P. patens*. Another probe for type II AGP, JIM13, labeled cell walls and cell plates in developing protoplasts, but did not label protonemal tips, rhizoids, or gametophore sections. These antibodies recognize different epitopes (Knox et al., 1991; Yates et al., 1996), so the divergent labeling patterns may reflect AGP structural heterogeneity. In previous studies LM6, a probe for pectic 1,5-α-L-arabinan, was shown to recognize *P. patens* AGP (Lee et al., 2005a,b). Together, the similar labeling patterns of LM2 and LM6 and the relatively weak anti-HG labeling of protonema and rhizoids, are consistent with recognition of AGP by LM6 and low pectin content in protonemal and rhizoid cell walls. Taken together, these results indicated that AGP is a major component of moss cell walls and may have specialized roles in protoplasts regeneration, tip growth, and differentiation of leaf midribs. The importance of AGPs in cell wall development in other bryophyte species is indicated by AGP immunogold labeling in various tissues of several bryophyte species (Ligrone et al., 2002) and by LM2 and JIM13 labeling of regenerating protoplasts and inhibition of protoplast division by Yariv reagent in *M. polymorpha* (Shibaya and Sugawara, 2009).

## Conclusion

Immuno and affinity histochemical labeling, along with flow cytometric analysis of regenerating protoplasts, has extended earlier glycan array analysis of *P. patens* cell walls to reveal spatial and developmental patterns of cell wall compositional variation in *P. patens*. These data provide a baseline for analyzing mutants for defects in cell wall structure and development.

## Author Contributions

EB carried out the protoplast and rhizoid immunofluorescence and helped coordinate preparation of the figures. MT carried out the flow cytometry analysis. CD and MB carried out the immunofluorescence analysis of protonema in response to osmotic stress. TS-D carried out the immunofluorescence analysis of gametophore sections. AR coordinated the project and the drafting of the manuscript. All authors participated in writing the manuscript and read and approved the final version.

## Conflict of Interest Statement

The authors declare that the research was conducted in the absence of any commercial or financial relationships that could be construed as a potential conflict of interest.

## References

[B1] Blake A. W. McCartney L. Flint J. E. Bolam D. N. Boraston A. B. Gilbert H. J. (2006). Understanding the biological rationale for the diversity of cellulose-directed carbohydrate-binding modules in prokaryotic enzymes. *J. Biol. Chem.* 281 29321–29329. 10.1074/jbc.M605903200 16844685

[B2] Carafa A. Duckett J. G. Knox J. P. Ligrone R. (2005). Distribution of cell-wall xylans in bryophytes and tracheophytes: new insights into basal interrelationships of land plants. *New Phytol.* 168 231–240. 10.1111/j.1469-8137.2005.01483.x 16159336

[B3] Chen X. Y. Kim J. Y. (2009). Callose synthesis in higher plants. *Plant Signal. Behav.* 4 489–492. 10.4161/psb.4.6.8359 19816126 PMC2688293

[B4] Cove D. (2005). The moss *Physcomitrella patens*. *Annu. Rev. Genet.* 39 339–358. 10.1146/annurev.genet.39.073003.110214 16285864

[B5] Cove D. J. Perroud P. F. Charron A. J. McDaniel S. F. Khandelwal A. Quatrano R. S. (2009). “The moss *Physcomitrella patens*. A novel model system for plant development and genomic studies,” in *Emerging Model Organisms: A Laboratory Manual*, eds Behringer R. R. Johnson A. D. Krumlauf R. E. (New York, NY: Cold Spring Harbor Laboratory Press), 69–104. 10.1101/pdb.emo115 20147063

[B6] Cuming A. C. Cho S. H. Kamisugi Y. Graham H. Quatrano R. S. (2007). Microarray analysis of transcriptional responses to abscisic acid and osmotic, salt, and drought stress in the moss, *Physcomitrella patens*. *New Phytol.* 176 275–287. 10.1111/j.1469-8137.2007.02187.x 17696978

[B7] Domozych D. S. Serfis A. Kiemle S. Gretz M. R. (2007). The structure and biochemistry of the homogalacturonans of the cell wall of the desmid, *Penium margaritaceum*. *Protoplasma* 230 99–115. 10.1007/s00709-006-0197-8 17111095

[B8] Eder M. Tenhaken R. Driouich A. Lutz-Meindl U. (2008). Occurrence and characterization of arabinogalactan-like proteins and hemicelluloses in *Micrasterias* (Streptophyta). *J. Phycol.* 44 1221–1234. 10.1111/j.1529-8817.2008.00576.x 27041719

[B9] Fu H. Yadav M. P. Nothnagel E. A. (2007). *Physcomitrella patens* arabinogalactan proteins contain abundant terminal 3-O-methyl-L-rhamnosyl residues not found in angiosperms. *Planta* 226 1511–1524. 10.1007/s00425-007-0587-y 17653569

[B10] Goss C. A. Brockmann D. J. Bushoven J. T. Roberts A. W. (2012). A CELLULOSE SYNTHASE (CESA) gene essential for gametophore morphogenesis in the moss *Physcomitrella patens*. *Planta* 235 1355–1367. 10.1007/s00425-011-1579-5 22215046

[B11] Gunning B. E. S. (1982). “The cytokinetic apparatus: its development and spatial regulation,” in *The Cytoskeleton in Plant Growth and Development*, ed LLoyd C. W. (London: Academic Press), 229–292.

[B12] Harholt J. Sørensen I. Fangel J. Roberts A. Willats W. G. T. Scheller H. V. (2012). The glycosyltransferase repertoire of the spikemoss *Selaginella moellendorffii* and a comparative study of its cell wall. *PLoS ONE* 7:e35846 10.1371/journal.pone.0035846 PMC3342304 22567114

[B13] Harkins K. R. Galbraith D. W. (1984). Flow sorting and culture of plant protoplasts. *Physiol. Plant.* 60 43–52. 10.1111/j.1399-3054.1984.tb04247.x 24253474

[B14] Hervé C. Marcus S. E. Knox J. P. (2011). “Monoclonal antibodies, carbohydrate-binding modules, and the detection of polysaccharides in plant cell walls,” in *The Plant Cell Wall: Methods and Protocols*, ed Popper Z. A. (New York, NY: Springer), 103–113. 10.1007/978-1-61779-008-9_7 21222079

[B15] Hiss M. Laule O. Meskauskiene R. M. Arif M. A. Decker E. L. Erxleben A. (2014). Large-scale gene expression profiling data for the model moss *Physcomitrella patens* aid understanding of developmental progression, culture and stress conditions. *Plant J.* 79 530–539. 10.1111/tpj.12572 24889180

[B16] Hornblad E. Ulfstedt M. Ronne H. Marchant A. (2013). Partial functional conservation of IRX10 homologs in *Physcomitrella patens* and *Arabidopsis thaliana* indicates an evolutionary step contributing to vascular formation in land plants. *BMC Plant Biol.* 13:3 10.1186/1471-2229-13-3 PMC3543728 23286876

[B17] Iraki N. M. Bressan R. A. Hasegawa P. M. Carpita N. C. (1989a). Alteration of the physical and chemical structure of the primary cell wall of growth-limited plant cells adapted to osmotic stress. *Plant Physiol.* 91 39–47. 10.1104/pp.91.1.39 16667031 PMC1061949

[B18] Iraki N. M. Singh N. Bressan R. A. Carpita N. C. (1989b). Cell walls of tobacco cells and changes in composition associated with reduced growth upon adaptation to water and saline stress. *Plant Physiol.* 91 48–53. 10.1104/pp.91.1.48 16667041 PMC1061950

[B19] Jensen J. K. Johnson N. R. Wilkerson C. G. (2014). *Arabidopsis thaliana* IRX10 and two related proteins from psyllium and *Physcomitrella patens* are xylan xylosyltransferases. *Plant J.* 80 207–215. 10.1111/tpj.12641 25139408

[B20] Jones L. Seymour G. B. Knox J. P. (1997). Localization of pectic galactan in tomato cell walls using a monoclonal antibody specific to (1[->]4)-[beta]-D-Galactan. *Plant Physiol.* 113 1405–1412. 12223681 10.1104/pp.113.4.1405 PMC158264

[B21] Knox J. P. (2008a). Mapping the walls of the kingdom: the view from the horsetails. *New Phytol.* 179 1–3. 10.1111/j.1469-8137.2008.02470.x 18557872

[B22] Knox J. P. (2008b). Revealing the structural and functional diversity of plant cell walls. *Curr. Opin. Plant Biol.* 11 308–313. 10.1016/j.pbi.2008.03.001 18424171

[B23] Knox J. P. Linstead P. J. Peart J. Cooper C. Roberts K. (1991). Developmentally regulated epitopes of cell surface arabinogalactan proteins and their relation to root tissue pattern formation. *Plant J.* 1 317–326. 10.1046/j.1365-313X.1991.t01-9-00999.x 21736649

[B24] Kremer C. Pettolino F. Bacic A. Drinnan A. (2004). Distribution of cell wall components in *Sphagnum* hyaline cells and in liverwort and hornwort elaters. *Planta* 219 1023–1035. 10.1007/s00425-004-1308-4 15290291

[B25] Kulkarni A. R. Peña M. J. Avci U. Mazumder K. Urbanowicz B. R. Pattathil S. (2012). The ability of land plants to synthesize glucuronoxylans predates the evolution of tracheophytes. *Glycobiology* 22 439–451. 10.1093/glycob/cwr117 22048859

[B26] Lee K. J. D. Knight C. D. Knox J. P. (2005a). *Physcomitrella patens*: a moss system for the study of plant cell walls. *Plant Biosys.* 139 16–19. 10.1080/11263500500055213

[B27] Lee K. J. D. Marcus S. E. Knox J. P. (2011). Cell wall biology: perspectives from cell wall imaging. *Mol. Plant* 4 212–219. 10.1093/mp/ssq075 21199879

[B28] Lee K. J. D. Sakata Y. Mau S.-L. Pettolino F. Bacic A. Quatrano R. S. (2005b). Arabinogalactan proteins are required for apical cell extension in the moss *Physcomitrella patens*. *Plant Cell* 17 3051–3065. 10.1105/tpc.105.034413 16199618 PMC1276029

[B29] Leroux O. Sorensen I. Marcus S. E. Viane R. L. Willats W. G. Knox J. P. (2015). Antibody-based screening of cell wall matrix glycans in ferns reveals taxon, tissue and cell-type specific distribution patterns. *BMC Plant Biol.* 15:56 10.1186/s12870-014-0362-8 PMC4351822 25848828

[B30] Liepman A. H. Nairn C. J. Willats W. G. T. Sørensen I. Roberts A. W. Keegstra K. (2007). Functional genomic analysis supports conservation of function among Cellulose synthase-like A gene family members and suggests diverse roles of mannans in plants. *Plant Physiol.* 143 1881–1893. 10.1104/pp.106.093989 17307900 PMC1851810

[B31] Ligrone R. Vaughn K. C. Renzaglia K. S. Knox J. P. Duckett J. G. (2002). Diversity in the distribution of polysaccharide and glycoprotein epitopes in the cell walls of bryophytes: new evidence for the multiple evolution of water-conducting cells. *New Phytol.* 156 491–508. 10.1046/j.1469-8137.2002.00538.x 33873570

[B32] Mansouri K. (2012). *Comparative Ultrastructure of Apical Cells and Derivatives in Bryophytes, With Special Reference to Plasmodesmata*. Carbondale, IL: Southern Illinois University, Department of Plant Biology.

[B33] Marcus S. E. Verhertbruggen Y. Hervé C. Ordaz-Ortiz J. J. Farkas V. Pedersen H. L. (2008). Pectic homogalacturonan masks abundant sets of xyloglucan epitopes in plant cell walls. *BMC Plant Biol.* 8:60 10.1186/1471-2229-8-60 PMC2409341 18498625

[B34] McCarthy T. W. Der J. P. Honaas L. A. dePamphilis C. W. Anderson C. T. (2014). Phylogenetic analysis of pectin-related gene families in *Physcomitrella patens* and nine other plant species yields evolutionary insights into cell walls. *BMC Plant Biol.* 14:79 10.1186/1471-2229-14-79 PMC4108027 24666997

[B35] McCartney L. Marcus S. E. Knox J. P. (2005). Monoclonal antibodies to plant cell wall xylans and arabinoxylans. *J. Histochem. Cytochem.* 53 543–546. 10.1369/jhc.4B6578.2005 15805428

[B36] Meikle P. J. Bonig I. Hoogenraad N. J. Clarke A. E. Stone B. A. (1991). The location of (1→3)-b-glucans in the walls of pollen tubes of *Nicotiana alata* using a (1→3)-b-glucan-specific monoclonal antibody. *Planta* 185 1–8. 10.1007/BF00194507 24186272

[B37] Menand B. Calder G. Dolan L. (2007). Both chloronemal and caulonemal cells expand by tip growth in the moss *Physcomitrella patens*. *J. Exp. Bot.* 58 1843–1849. 10.1093/jxb/erm047 17404383

[B38] Mishler B. D. Oliver M. J. (2009). “Putting *Physcomitrella patens* on the tree of life: the evolution and ecology of mosses,” in *The Moss Physcomitrella patens*, eds Knight C. D. Perroud P. F. Cove D. (Chichester: Blackwell), 1–15.

[B39] Moller I. Sørensen I. Bernal A. J. Blaukopf C. Lee K. Øbro J. (2007). High-throughput mapping of cell-wall polymers within and between plants using novel microarrays. *Plant J.* 50 1118–1128. 10.1111/j.1365-313X.2007.03114.x 17565618

[B40] Nishiyama T. Fujita T. Shin-I T. Seki M. Nishide H. Uchiyama I. (2003). Comparative genomics of *Physcomitrella patens* gametophytic transcriptome and *Arabidopsis thaliana*: implication for land plant evolution. *Proc. Natl. Acad. Sci. U.S.A.* 100 8007–8012. 10.1073/pnas.0932694100 12808149 PMC164703

[B41] Pattathil S. Avci U. Baldwin D. Swennes A. G. McGill J. A. Popper Z. (2010). A comprehensive toolkit of plant cell wall glycan-directed monoclonal antibodies. *Plant Physiol.* 153 514–525. 10.1104/pp.109.151985 20363856 PMC2879786

[B42] Peña M. J. Darvill A. G. Eberhard S. York W. S. O’Neill M. A. (2008). Moss and liverwort xyloglucans contain galacturonic acid and are structurally distinct from the xyloglucans synthesized by hornworts and vascular plants. *Glycobiology* 18 891–904. 10.1093/glycob/cwn078 18703646

[B43] Pettolino F. A. Hoogenraad N. J. Ferguson C. Bacic A. Johnson E. Stone B. A. (2001). A (1–>4)-beta-mannan-specific monoclonal antibody and its use in the immunocytochemical location of galactomannans. *Planta* 214 235–242. 10.1007/s004250100606 11800387

[B44] Popper Z. A. (2008). Evolution and diversity of green plant cell walls. *Curr. Opin. Plant Biol.* 11 286–292. 10.1016/j.pbi.2008.02.012 18406657

[B45] Rensing S. A. Lang D. Zimmer A. D. Terry A. Salamov A. Shapiro H. (2008). The *Physcomitrella* genome reveals evolutionary insights into the conquest of land by plants. *Science* 319 64–69. 10.1126/science.1150646 18079367

[B46] Richardt S. Timmerhaus G. Lang D. Qudeimat E. Correa L. G. Reski R. (2010). Microarray analysis of the moss *Physcomitrella patens* reveals evolutionarily conserved transcriptional regulation of salt stress and abscisic acid signalling. *Plant Mol. Biol.* 72 27–45. 10.1007/s11103-009-9550-6 19806323

[B47] Roberts A. W. Budziszek M. J. Dimos C. S. Goss C. A. Lai V. (2011). Knocking out the wall: protocols for gene targeting in *Physcomitrella patens*. *Methods Mol. Biol.* 715 273–290. 10.1007/978-1-61779-008-9_19 21222091

[B48] Roberts A. W. Bushoven J. T. (2007). The cellulose synthase (CESA) gene superfamily of the moss *Physcomitrella patens*. *Plant Mol. Biol.* 63 207–219. 10.1007/s11103-006-9083-1 17006591

[B49] Roberts A. W. Roberts E. M. Haigler C. H. (2012). Moss cell walls: structure and biosynthesis. *Front. Plant Sci.* 3:166 10.3389/fpls.2012.00166 PMC3400098 22833752

[B50] Sakakibara K. Nishiyama T. Sumikawa N. Kofuji R. Murata T. Hasebe M. (2003). Involvement of auxin and a homeodomain-leucine zipper I gene in rhizoid development of the moss *Physcomitrella patens*. *Development* 130 4835–4846. 10.1242/dev.00644 12917289

[B51] Samuels A. L. Giddings T. H. Jr. Staehelin L. A. (1995). Cytokinesis in tobacco BY-2 and root tip cells: a new model of cell plate formation in higher plants. *J. Cell Biol.* 130 1345–1357. 10.1083/jcb.130.6.1345 7559757 PMC2120572

[B52] Schuette S. Wood A. J. Geisler M. Geisler-Lee J. Ligrone R. Renzaglia K. S. (2009). Novel localization of callose in the spores of *Physcomitrella patens* and phylogenomics of the callose synthase gene family. *Ann. Bot.* 103 749–756. 10.1093/aob/mcn268 19155219 PMC2707875

[B53] Shea E. M. Gibeaut D. M. Carpita N. C. (1989). Structural analysis of the cell walls regenerated by carrot protoplasts. *Planta* 179 293–308. 10.1007/BF00391074 24201658

[B54] Shibaya T. Sugawara Y. (2009). Induction of multinucleation by beta-glucosyl Yariv reagent in regenerated cells from *Marchantia polymorpha* protoplasts and involvement of arabinogalactan proteins in cell plate formation. *Planta* 230 581–588. 10.1007/s00425-009-0954-y 19475420

[B55] Skirycz A. De Bodt S. Obata T. De Clercq I. Claeys H. De Rycke R. (2010). Developmental stage specificity and the role of mitochondrial metabolism in the response of *Arabidopsis* leaves to prolonged mild osmotic stress. *Plant Physiol.* 152 226–244. 10.1104/pp.109.148965 19906889 PMC2799359

[B56] Smallwood M. Yates E. A. Willats W. G. T. Martin H. Knox J. P. (1996). Immunochemical comparison of membrane-associated and secreted arabinogalactan-proteins in rice and carrot. *Planta* 198 452–459. 10.1007/BF00620063

[B57] Sørensen I. Pettolino F. A. Bacic A. Ralph J. Lu F. O’Neill M. A. (2011). The Charophycean green algae provide insights into the early origins of plant cell walls. *Plant J.* 68 201–211. 10.1111/j.1365-313X.2011.04686.x 21707800

[B58] Uhnak K. S. Roberts A. W. (1995). Microtubule rearrangements accompanying dedifferentiation in mesophyll cultures of *Zinnia elegans* L. *Protoplasma* 189 81–87. 10.1007/BF01280293

[B59] van Amstel T. N. M. Kengen H. M. P. (1996). Callose deposition in the primary wall of suspension cells and regenerating protoplasts, and its relationship to patterned cellulose synthesis. *Can. J. Bot.* 74 1040–1049. 10.1139/b96-128

[B60] Verhertbruggen Y. Marcus S. E. Haeger A. Ordaz-Ortiz J. J. Knox J. P. (2009). An extended set of monoclonal antibodies to pectic homogalacturonan. *Carbohydr. Res.* 344 1858–1862. 10.1016/j.carres.2008.11.010 19144326

[B61] Wang T. McFarlane H. E. Persson S. (2016). The impact of abiotic factors on cellulose synthesis. *J. Exp. Bot.* 67 543–552. 10.1093/jxb/erv488 26552883

[B62] Willats W. G. Marcus S. E. Knox J. P. (1998). Generation of monoclonal antibody specific to (1–>5)-alpha-L-arabinan. *Carbohydr. Res.* 308 149–152. 10.1016/S0008-6215(98)00070-6 9675359

[B63] Willats W. G. T. Knox J. P. (2003). Molecules in context: probes for cell wall analysis. *Ann. Plant Rev.* 8 92–110.

[B64] Wise H. Z. Saxena I. M. Brown R. M. Jr. (2011). Isolation and characterization of the cellulose synthase genes PpCesA6 and PpCesA7 in *Physcomitrella patens*. *Cellulose* 18 371–384. 10.1007/s10570-010-9479-6

[B65] Xue J. Bosch M. Knox J. P. (2013). Heterogeneity and glycan masking of cell wall microstructures in the stems of *Miscanthus* x giganteus, and its parents *M. sinensis* and *M. sacchariflorus*. *PLoS ONE* 8:e82114 10.1371/journal.pone.0082114 PMC3843723 24312403

[B66] Yates E. A. Valdor J. F. Haslam S. M. Morris H. R. Dell A. Mackie W. (1996). Characterization of carbohydrate structural features recognized by anti-arabinogalactan-protein monoclonal antibodies. *Glycobiology* 6 131–139. 10.1093/glycob/6.2.131 8727785

[B67] Yin Y. Chen H. Hahn M. G. Mohnen D. Xu Y. (2010). Evolution and function of the plant cell wall synthesis-related glycosyltransferase family 8. *Plant Physiol.* 153 1729–1746. 10.1104/pp.110.154229 20522722 PMC2923890

[B68] Yin Y. Huang J. Xu Y. (2009). The cellulose synthase superfamily in fully sequenced plants and algae. *BMC Plant Biol.* 9:99 10.1186/1471-2229-9-99 PMC3091534 19646250

[B69] Zimmer A. D. Lang D. Buchta K. Rombauts S. Nishiyama T. Hasebe M. (2013). Reannotation and extended community resources for the genome of the non-seed plant *Physcomitrella patens* provide insights into the evolution of plant gene structures and functions. *BMC Genomics* 14:498 10.1186/1471-2164-14-498 PMC3729371 23879659

